# The Use of HRP in Decolorization of Reactive Dyes and Toxicological Evaluation of Their Products

**DOI:** 10.4061/2010/703824

**Published:** 2011-01-20

**Authors:** Michelle Reis da Silva, Lívian Ribeiro Vasconcelos de Sá, Carlos Russo, Elita Scio, Viridiana Santana Ferreira-Leitão

**Affiliations:** ^1^Biocatalysis Laboratory, Catalysis Division, National Institute of Technology, Ministry of Science and Technology, Avenue Venezuela 82, Sala 302, 20081-312 Rio de Janeiro, RJ, Brazil; ^2^Department of Technology and Biochemical Processes, Chemistry Institute, State University of Rio de Janeiro, Rua São Francisco Xavier, 524, Lab 310, 20550-900 Rio de Janeiro, RJ, Brazil; ^3^Department of Biochemistry, Federal University of Juiz de Fora, 36036-330 Juiz de Fora, MG, Brazil

## Abstract

This work studied the potential use of *horseradish peroxidase* (HRP) in the decolorization of the following textile dyes: Drimarene Blue X-3LR (DMBLR), Drimarene Blue X-BLN (DMBBLN), Drimarene Rubinol X-3LR (DMR), and Drimarene Blue CL-R (RBBR). Dyes were individually tested in the reaction media containing 120 mg·L^−1^, considering the following parameters: temperature (20–45°C), H_2_O_2_ concentration (0–4.44 mmol·L^−1^), and reaction time (5 minutes, 1 and 24 h). The following conditions: 35°C, 0.55 mmol·L^−1^, and 1h, provided the best set of results of color removal for DMBLR (99%), DMBBLN (77%), DMR (94%), and RBBR (97%). It should be mentioned that only 5 minutes of reaction was enough to obtain 96% of decolorization for DMBLR and RBBR. After the decolorization reactions of DMBLR, DMR, and RBBR, it was possible to observe the reduction of *Artemia salina* mortality and the no significant increase in toxicity for the products generated from DMBBLN.

## 1. Introduction

The textile industry is a large consumer of energy and potable water, mainly after dyeing in the washing procedures. During industrial processing, up to 40% of the used dyestuffs are released in the effluent. Considering the high discharged volumes and their composition, wastewater from textile industries can be considered as one of the most polluting in all industrial sectors, thus requiring appropriate treatment technologies [1]. The removal of color from effluent is one of the major problems that the textile industry faces. The presence of color hinders the absorption of solar radiation, thus reducing the natural photosynthetic activity, causing changes in aquatic biota. Moreover, many of these dyes present acute or chronic toxicity on the ecosystems [2].

When compared to natural dyes, reactive dyes are extensively used in the textile industry due to their easy use, cost effectiveness in synthesis, stability, variety of colors, and better dyeing processing conditions [3–5]. On the other hand, many synthetic dyes are resistant to biological degradation due to the presence of large content of aromatic structure, complex molecules, and synthetic origin; thus, the decolorization of textile dye effluent does not occur when treated in conventional effluent treatment systems [3, 6, 7]. Different techniques of color removal have been studied, such as adsorption, precipitation, oxidation, coagulation-flocculation, chemical degradation and photodegradation. These methods have different color removal capabilities and capital costs. Coagulation and adsorption are the most commonly used but generate large amounts of solid wastes and subsequent disposal problems. There is a great need to develop an effective way of dealing with textile dye effluent [6].

Many authors are focusing their attention on enzymatic treatment of the synthetic dyes [8–16], which should be used in association with conventional treatments. Enzymatic treatment can be used in a complementary manner in accordance with the following characteristics: application to recalcitrant materials, operation at high and low contaminant concentration over wide pH, temperature, and salinity ranges [17–20]. Several works have demonstrated that extracellular enzymes from white rot fungi such as peroxidases (*lignin peroxidase* (LiP) and *manganese peroxidase* (MnP)) and phenoloxidases (laccases) can be used to degrade and detoxify polyaromatic hydrocarbons, polychlorinated biphenyls, and other dyes [8, 13, 21, 22]. Plant peroxidases, such as horseradish peroxidase-HRP, also deserve attention for environmental applications. Previous studies showed interesting results with dyes and other xenobiotics [10, 23–25].

The enzyme *horseradish peroxidase* (HRP) has been successfully used in diagnostic kits of medical interest. This enzyme is also well known for its effective capacity to oxidize a wide spectrum of aromatic compounds, as well as in the degradation of some important industrial dyes [12, 23, 26]. In Brazil, this enzyme is produced by Toyobo-Brazil, which renders significant cost reductions in the enzymatic process, due to high cost of importation. In addition, new applications for this traditional enzyme have been researched, aiming to expand the market for this enzyme.

The efficiency of the enzymatic treatment of dyes should also consider the formation of toxic products during the color removal process; thus, the use of bioindicators to evaluate the toxicity formed may confirm its effectiveness [23, 27]. This work studied the potential use of *horseradish peroxidase* enzyme in decolorization of the textile dyes widely used in the Brazilian textile industry, considering the following parameters: temperature, H_2_O_2_ concentration, and reaction time. The toxicity of the dyes and their degradation products after enzyme treatment was also evaluated using *Artemia salina*, as bioindicator.

The most interesting point of this work lies in the use of an enzyme produced in Brazil to degrade dyes used in the Brazilian industry. Therefore, this study proposes a solution for an environmental problem as well as promotes the expansion of the domestic market for this enzyme, in other words, a tailor-made solution. Considering the difficulties to obtain real effluents to test new methodologies for dyes treatment, the high efficiency in colour removal using synthetic media and toxicity minimization reinforce the potential use of HRP with environmental purpose.

## 2. Materials and Methods

### 2.1. Dyes

**Figure 1:**
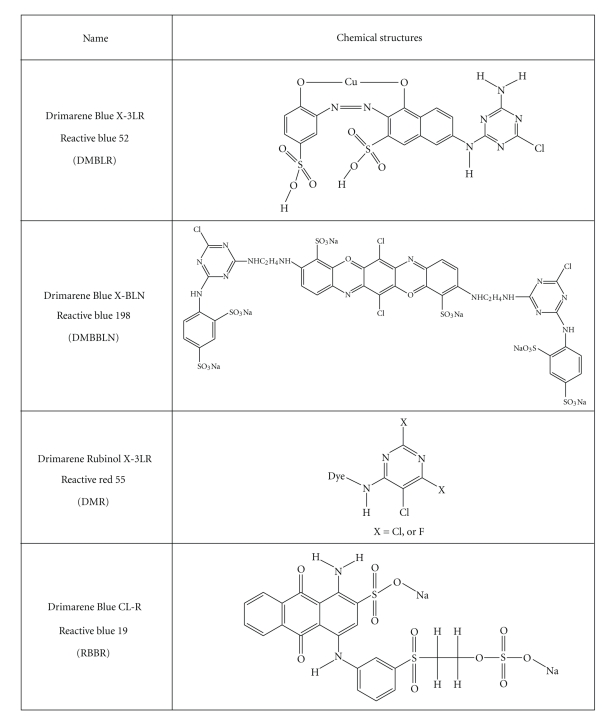
Chemical structures of the dyes DMBLR, DMBBLN, DMR e RBBR [33–35].

The dyes studied were Drimarene Blue X-3LR (DMBLR), Drimarene Blue X-BLN (DMBBLN), Drimarene Rubinol X-3LR (DMR), and Drimarene Blue CL-R (RBBR). Dyes solutions were prepared with distilled water. Chemical structures of the dyes DMBLR, DMBBLN, DMR and RBBR are presented in Figure 1. 

The textile dyes were provided by Maria Cândida Textile Industry LTDA, Paracambi, Rio de Janeiro, Brazil.

### 2.2. Horseradish Peroxidase (HRP)

 In this study, the peroxidase used was *horseradish peroxidase* (HRP) provided by Toyobo-Brazil LTDA. The enzyme (70 *μ*g·mL^−1^) was prepared in sodium phosphate buffer 0.2 mol·L^−1^ (pH 6.0) and stored at 4°C.

The enzymatic activity was determined through the oxidation reaction of 2,4-dichlorofenol (2,4-DCP) in the presence of 4-aminoantipyrine (4-AAP) resulting in the formation of the colored compound antipirilquinonimine. Reactions were followed by spectrophotometric analysis, at 510 nm (*ξ* = 18.500 M^−1^cm^−1^). One unit of enzyme activity was defined as the amount of enzyme able to oxidize 1 *μ*mol of substrate per minute. The HRP activity was maintained at 3.5 U·mL^−1^ during all the experiments.

### 2.3. Decolorization Assay

Reaction media contained dye solutions in the concentration of 120 mg·L^−1^, sodium phosphate buffer solution (pH 6.0) 0.083 mol·L^−1^, enzyme activity of 3.5 U·mL^−1^, and H_2_O_2_ concentration of (0–4.44 mmol·L^−1^). Experiments were carried out in five different temperatures (20, 25, 35, 40 and 45°C) and monitored for 5 minutes, 1 and 24 h. Control experiments were employed in absence of H_2_O_2_. All tests were carried out in triplicate.

Decolorization efficiencies were determined by absorbance readings, before and after reaction, at the maximum wavelengths, which were determined for each dye in the reaction media: 616 nm (DMBLR), 626 nm (DMBBLN), 530 nm (DMR), and 602 nm (RBBR), using a HACH DR/4000 spectrophotometer.

### 2.4. Acute Toxicity Test with Artemia salina

*Artemia salina* was used as bioindicator since the effluent from the textile industries has high salinity and, therefore, high conductivity, which makes this a critical parameter for freshwater species [28].

The acute toxicity tests were carried out according to the methodology developed by Meyer and coworkers in 1982 [29] and modified by Neto in 2003 [30]. Larvae of brine shrimp (*Artemia salina*) were obtained after eclosion from dry eggs in artificial sea water with aeration for 48 h. Subsequently, the larvae were transferred to each set of tubs (10 larvae/tub) with different concentrations of reaction media containing the dyes or their degradation products after enzymatic treatment. After 24 h, the number of survivors was counted. The percentage of mortality of *Artemia salina* was related to the concentrations of reaction media before and after enzymatic treatment of dyes. The following concentrations of reaction media were used: 100, 90, 80, 70, 60, 50, 40, 30, 20, and 10% (v/v). Both positive (thymol at 120 mg·L^−1^) and negative (artificial sea water) control assays were performed in parallel. Tests were carried out in triplicate.

## 3. Results and Discussion

### 3.1. Temperature

**Figure 2:**
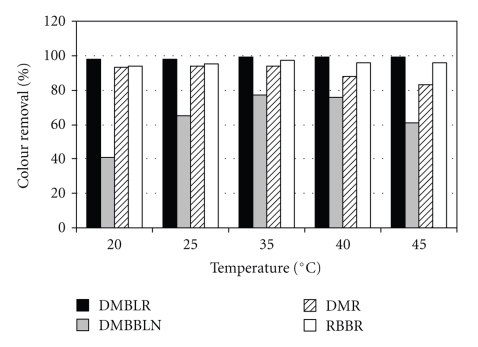
Influence of temperature on the decolorization of textile dyes. Reaction conditions: enzyme's activity = 3.5 U·mL^−1^, dyes concentrations = 120 mg·L^−1^, H_2_O_2_ = 0.55 mmol·L^−1^, and reaction time = 1 h.

Temperature effect on dyes decolorization mediated by HRP was studied from 20°C, up to 45°C and the results are presented in Figure 2. The best result of dye decolorization was achieved at 35°C: 99% DMBLR, 77% DMBBLN, 94% DMR, and 97% RBBR.

Our results also showed that the decolorization of DMBLR and RBBR was not affected by the variation of temperature, maintaining the high percentage of decolorization in all cases (Figure 2). The opposite behavior was observed for DMBBLN and DMR. At 45°C, DMBBLN presented a reduction of 15% and DMR 10%, when compared to the result obtained at 35°C. Previous studies reported that laccase activity was not affected by temperature increase during the decolorization tests of DMBLR [13]. The study of Methylene blue decolorization by *lignin peroxidase* and HRP also reported enzyme activity resistance at high temperatures [22].

Contrary to what was previously reported, it was possible to verify a reduction of 50% in the efficiency of the decolorization of the dyes Bromophenol and Mehyl orange by HRP, at 40°C in a range from 30 to 80°C [14]. This behavior could be related to the loss of enzymatic activity at high temperatures, also observed in other studies [14, 24, 31]. 

The different observations concerning the effect of temperature on enzyme activity in the degradation reactions can be explained by the known relationship between the thermal deactivation of enzymes and the presence of some phenomena that tend to increase the speed of reaction in higher temperatures [25].

### 3.2. H_2_O_2_ Concentration

**Figure 3:**
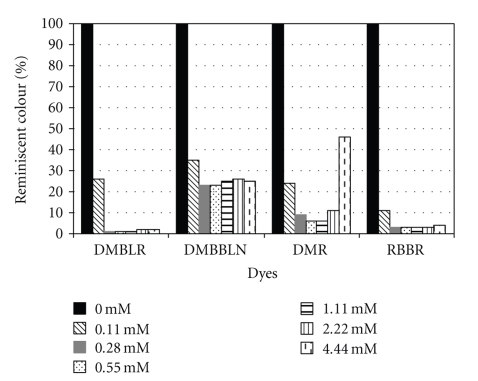
Influence of H_2_O_2_ concentration on the decolorization of textile dyes. Reaction conditions: enzyme's activity = 3.5 U·mL^−1^, dyes concentrations = 120 mg·L^−1^, temperature = 35°C and, reaction time = 1 h.

Figure 3 shows the percentage of remaining color in the absence of H_2_O_2_, which shows the importance and necessity of an appropriated H_2_O_2_ concentration. In this study, was also observed that low H_2_O_2_ concentrations hinder the enzyme action, and the excess of this reagent causes enzyme inactivation. Similar results were also reported in previous studies that emphasize the susceptibility of the peroxidase to high H_2_O_2_ concentrations [12, 22, 23, 25, 31, 32]. The best enzyme performance was observed in the H_2_O_2_ concentration of 0.55 mmol·L^−1^, where the molar ratios between dye and H_2_O_2_ were 1 : 3, 1 : 7, and 1 : 3, respectively, for the dyes DMBLR, DMBBLN, and RBBR. This condition was efficient to remove 99% color of DMBLR, 77% DMBBLN, 94% DMR, and 97% RBBR with dye concentrations of 120 mg·L^−1^. The molar ratio of the DMR was not calculated because of its undefined molecular structure.

### 3.3. Reaction Time

**Figure 4:**
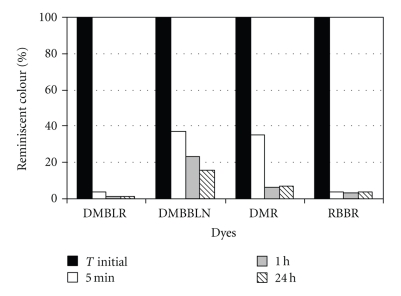
Influence of reaction time on the decolorization of textile dyes. Reaction conditions: enzyme's activity = 3.5 U·mL^−1^, dyes concentrations = 120 mg·L^−1^, temperature = 35°C, and H_2_O_2_ concentration = 0.55 mmol·L^−1^.

HRP showed a high efficiency in dye decolorization in a short time of reaction (Figure 4). Very promising results were obtained in only 5 minutes of reaction. DMBLR and RBBR showed 96% of color removal. After 1 h of reaction, dyes' decolorization was improved, showing the following percentages: 99% DMBLR, 77% DMBBLN, 94% DMR, and 97% RBBR. An increase of less than 10% in the percentage of DMBBLN decolorization was observed after 24 h of reaction (84% of decolorization). 

Only 5 minutes were enough to obtain the degradation of the dyes Bromophenol and Methyl orange, with decolorization of 100 and 80%, respectively, by citraconic anhydride-modified HRP [14]. However, studies had reported that 8 and 10 h were the periods of time required to catalyze the enzymatic decolorization of the dyes DMBLR and RBBR, respectively, at 100 mg·L^−1^ by *Funalia trogii *[33]. The same dyes were studied in this work in the concentration of 120 mg·L^−1^, and 96% of decolorization was obtained for both dyes in only 5 minutes of reaction. In other words, this study achieved better yields using higher concentrations.

Souza *and coworkers in *2007 [23] studied the decolorization of different dyes, and they observed that the reaction time is directly related to the different structures of the dyes; this fact affected the way of enzyme activity and consequently could cause variation in the reaction time. 

The efficiency of the enzyme HRP in the decolorization of RBBR in this work was compared to other previous studies reported in the literature [7, 8, 21, 33].  Table 1 presents comparative results emphasizing the high percentage of decolorization obtained using HRP as catalyst for enzymatic treatment of RBBR in 5 minutes, where 96% of decolorization was observed. The difference in the decolorization efficiency can be associated with the structural properties of each dye and with the specificity of the enzyme and substrate [1].

The effects of the DMBLR concentration on the color removal efficiency using enzyme complex from *Funalia trogii* were also studied [13]; 78% of decolorization was reached at 60 mg·L^−1^ in 2 minutes. In this work, 96% of decolorization of the same dye was obtained in a concentration 2 times higher (120 mg·L^−1^) in 5 minutes. Özsoy and coworkers [33] studied the enzymatic treatment of the dyes DMBLR and RBBR in 2005, both at the concentration of 100 mg·L^−1^ and observed 92 and 90% of decolorization after treatment by *Funalia trogii* in 8 and 10 h, respectively. 

The high percentage of decolorization achieved to DMBLR and RBBR at the concentrations above called attention to the efficiency of the enzymatic treatment of both dyes in this work when compared to the previous.

### 3.4. Toxicity Tests

The toxicity was evaluated before and after enzymatic treatment, aiming at verifying the efficiency of the HRP as environmental biocatalyst. A promising environmental biocatalyst should promote the color removal and the toxicity reduction.

**Figure 5:**
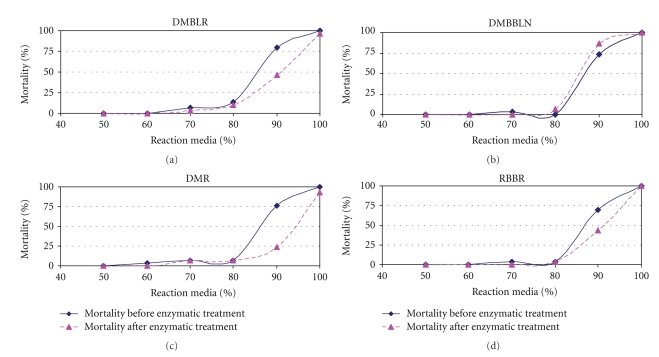
Percentage of mortality of *Artemia salina* according to the concentration of reaction media containing each dye—DMBLR, DMR, RBBR, and DMBBLN—before enzymatic action or their respective products after the enzymatic treatment with HRP.

The results are presented in Figure 5. The reduction of toxicity after enzymatic treatment of these dyes DMBLR, DMR and RBBR was confirmed by the low percentage of mortality of *Artemia salina, *namely, 47, 25 and 45% in the presence of 90% of the reaction media. Only the dye DMBBLN showed a small increase in the percentage of mortality of *Artemia salina* (10% higher in the presence of 90% of the reaction media), indicating a small increase in toxicity after treatment with HRP.

Souza and coworkers in 2007 [23] observed a low reduction in toxicity for the textile effluent after enzymatic treatment with HRP. In this work, it was shown that HRP was efficient in the decolorization of textile dyes as well as for achieving a reduction in the toxicity of the dyes DMBLR, DMR, and RBBR after the enzymatic treatment.

## 4. Conclusions

In this study, the enzyme HRP showed a promising performance as biocatalyst in the decolorization of textile dyes. The highest percentages of decolorization were observed for DMBLR and RBBR in 5 minutes of reaction. The same was not observed for DMR and DMBBLN, which seems to indicate that the enzyme would have less affinity for these dyes, directly reflected in the reaction time.

The acute toxicity tests for the textile dyes with *Artemia salina* showed that there was a toxicity reduction of reaction media of the dyes DMBLR, DMR, and RBBR after treatment with HRP. However, the toxicity of the reaction media containing DMBBLN dye was slightly, not significantly, higher after treatment with HRP. According to the preliminary results of toxicity, it is possible to conclude that the enzyme HRP was efficient in the decolorization, and the toxicity of the products formed was not a problem for this kind of treatment.

All results showed the potential use of HRP in the treatment of effluents containing reactive dyes, and as mentioned before, this work reinforces the use of a tailor-made solution for an environmental problem and also promoting the expansion of internal market for HRP.

## Figures and Tables

**Table 1:** Comparison of the enzymatic treatments used in the decolorization of RBBR.

Enzymatic treatments	Source	RBBR (mg·L^−1^)	Temperature (°C)	Reaction time (min)	Decolorization (%)	References
MnP and laccase	*Irpex lacteus*	150	28	8640	100	[8]
Laccase	*Ganoderma lucidum*	50	60	1200	90	[7]
Laccase + HBT				120	92	
Ligninolytic enzymes	Litter-decomposing fungi	100	25	40320	80–98	[21]
Enzymatic complex	*Funalia trogii*	100	30	600	90	[33]

Plant peroxidase (HRP)	*Armoracia rusticana*	120	35	60	97	Present work
				5	96	

RBBR: Drimarene Blue CL-R; MnP: manganese peroxidase; HBT: N-hydroxybenzotriazole; HRP: horseradish peroxidase.
